# Case of Artificial Anus, Cured by an Operation on the Principle of Tagliacozzi

**Published:** 1820-06

**Authors:** Geo. Fred. Collier

**Affiliations:** Surgeon, &c.


FOR THE LONDON MEDICAL AND PHYSICAL JOURNAL.

### *Case of Artificial Anus, cured by an Operation on the Principle of Tagliacozzi.* By GEO. FRED. COLLIER, Esq. Surgeon, &c.

THE following case I have drawn up, and now send to you for insertion in your Journal, at the request of some of my surgical brethren, who have done me the honour to give me credit for some originality and ingenuity in the cure of it.

Mr. A. requested me to see a servant of his, who, he said, had an aperture in the right groin, from which feces were occasionally voided. The man had, according to his own account, a swelling which came suddenly in the right groin, and which the *Caleb Quotem* of his parish called an abscess, and which he lanced: no matter escaped, although he plunged the lancet in very deep; and he left it, saying it was not yet ripe. The patient suffered much, and had incessant vomiting for some hours after, when at last the tumor became discoloured, and sloughed, leaving an aperture in which he could put his two thumbs: through this feces escaped; and, although the wound granulated and closed considerably, yet it left a fistulous, or rather, perhaps I should say, a contracted callous orifice, constituting an artificial anus.

When I first saw him, he had been in this way between two and three months. My first object was to destroy the callous edge, and produce healthy granulation: this failed, although conducted in the manner recommended by Mr. Cooper, in his treatment of fistulous sores in the urethra. My next attempt was to produce union by ligature: this failed, and produced some degree of inflammation. My last, and most successful effort, was to try the Tagliacozzian operation. Having first removed the callous edge of the aperture, and thus obtained an uniting surface of about twice the diameter of the orifice itself, I dissected off an adequate portion of integument immediately above it, preserving a small attachment to the original site, and then approximated it to the exposed surface by means of four sutures; having first twisted the flap so as to have the skin outermost. On this I applied a compress, and over it a truss. Complete union took place in the ordinary way, without one unpleasant symptom. Perhaps it is right to observe, that, before the operation, the feces were voided chiefly by the artificial anus when the bowels were constipated; but, *vice versa*, or when under the action of cathartics, almost all came away by the natural passage.

I would ask the following questions to any of your correspondents:

*Query* 1.—Was this operation ever attempted before?

2. Does any person consider it attended with danger, and under what circumstances?

And, lastly, if not so, why should it not become a general practice?

A drawing of the case may be seen at my house.

20, *Norfolk-street, London;*
*May* 11, 1820.

